# Affective and Social Competencies of Elementary School Students in the Use of Digital Textbooks: A Longitudinal Study

**DOI:** 10.3390/bs14030179

**Published:** 2024-02-25

**Authors:** Hyunjung Im

**Affiliations:** Department of Teaching Education, Dankook University, Yongin 16890, Republic of Korea; doongry@dankook.ac.kr

**Keywords:** digital textbook, e-learning, self-efficacy, social competence, longitudinal study

## Abstract

This study investigates the impact of digital textbooks, which play an important role in post-COVID-19 digitalized education, on the development of elementary school students’ affective and social competencies. The study quantitatively analyzed three years of student panel data collected from 1,418 students in the third and fourth grades of a digital textbook pilot elementary school in South Korea. This study examined differences in the development of affective and social competencies between the treatment group (*n* = 708), who used digital textbooks for three years, and the control group (*n* = 710), who used digital textbooks for two years and returned to paper textbooks. Results showed that the affective competencies of self-efficacy, learning motivation, and learning attitudes were higher in the treatment group than in the control group, and the social competencies of communication, collaboration, and sociability were also higher. Implications for digital education research and related policies are provided, and limitations and suggestions for future research are discussed.

## 1. Introduction

With the advancement of digital technology, an era has arrived where information and data can be shared more efficiently and effectively. In traditional media, books were a primary source of information, and they still maintain their significance in the digital age by adapting through digitalization. In the field of education, various methods have been actively researched to integrate digital technology into the educational environment and deliver positive effects. Particularly, as textbooks are the most critical learning medium in educational and learning environments, digital textbooks are one of the central issues in the digitalization of education [1,2].

The digital textbook is a concept that is developing dynamically and is currently expanding. The initial digitalization of textbooks referred to the simple transition from printed books to digital forms like e-books or static PDF files. Reflecting various demands in the educational field, they have evolved into interactive digital textbooks with added multimedia and interactive features [3,4]. Digital textbooks are notable for their flexibility in use without location constraints, their ability to quickly adapt and change according to the evolving needs of users [5], and for being a sustainable element of education due to their potential to save economic and environmental costs [6]. 

The impact of digital textbooks on students’ cognitive abilities and academic achievement has been vigorously researched since their early development. As enhancing cognitive skills has traditionally been a primary goal of school education, the effectiveness of digital textbooks has been extensively studied from a cognitive perspective. Numerous studies have reported enhancements in students’ cognitive abilities across various subjects when using digital textbooks [1,7,8]. Encouraged by these findings, many countries have initiated national policies to adopt digital textbooks nationwide [9].

Recently, education’s objectives have expanded to include not only cognitive aspects but also various aspects like social, affective, and civic competencies, reflecting a broader perspective of what education should encompass. This approach aligns with the principles of ESD (Education for Sustainable Development), which emphasizes the sustainability of education [10]. Particularly post-COVID, as education swiftly moved online, there has been significant concern over the decline in students’ social skills. This shift has sparked increased interest in developing communication, collaboration, and social skills so students can effectively engage in societal tasks regardless of their environment [11]. Concurrently, the remote learning environment has spurred research to keep students motivated and engaged [12], thus increasing focus on affective competencies like intrinsic motivation and self-efficacy. As the era evolves so does the role of school education. Despite the broadening scope of educational goals, the impact of digital textbooks on areas beyond cognition, notably emotional and social aspects, has received relatively less attention.

While digital textbooks are recognized for their advantages, concerns about their limitations remain prevalent. The efficacy of digital textbooks as replacements for traditional printed textbooks remains uncertain [13], and some studies have indicated a low student preference for digital textbooks [14]. Moreover, a common concern from a policy perspective is that of the substantial initial investment required for the implementation of digital textbooks [15].

Therefore, when considering the application of digital textbooks on a large or national scale, it is essential to make decisions carefully by evaluating the cost-effectiveness ratio. Decisions should especially be informed by evidence gathered from long-term longitudinal studies, and policies should proceed with a phased approach for wider application. However, reviews of the current literature on digital textbooks reveal that while many studies systematically examine the effectiveness of digital textbooks, there is a lack of long-term longitudinal studies [7,16]. Thus, the need for longitudinal studies on the practical and empirical effects of using digital textbooks in education is emphasized. The longitudinal study can provide great insights into the need to introduce digital textbooks and the design and modification of policies.

Based on this necessity, this study aimed to verify the effects of digital textbooks on the affective and social domains by analyzing large-scale data collected over three years. In particular, it examined the unique effects of digital textbooks by comparing a group that consistently used digital textbooks for three years with a group that switched back to printed textbooks midway. The research hypotheses are as follows:

**Hypothesis** **1 (H1).** *There will be a difference in the change in affective domain competencies (self-efficacy, learning motivation, and learning attitude) between the group that continued using digital textbooks and the group that stopped using them.*

**Hypothesis** **2 (H2).** *There will be a difference in the change in social domain competencies (communication skills, collaboration skills, and sociality) between the group that continued using digital textbooks and the group that discontinued their use.*

This study provides an empirical understanding of the impact of the use of digital textbooks on changes in students’ affective and social competencies. These findings contribute to efforts towards evidence-based policymaking by providing a basis for policy decisions related to digital textbooks, which in turn contribute to the improvement of students’ affective and social competencies. 

## 2. Literature Review

### 2.1. Definition and Development of Digital Textbooks

Digital textbooks should be viewed as a continuously evolving concept [17]. Initially, they were merely simple e-books like PDF files, shifting from a printed format to a digital format. However, as they were applied in educational settings and evolved to reflect the diverse needs of users, the current digital textbooks with various functionalities were developed. In recent digital textbooks, user interaction with the textbook has been emphasized, including features such as bookmarks, highlights, notes, hyperlinks, and access to external learning resources. They have even advanced into collaborative digital textbooks that include features for user interaction [7,18].

Ref. [3] categorizes digital textbooks into two generations. According to this classification, first-generation digital textbooks refer to those that have minimal interaction and are essentially digital versions of existing textbooks, often referred to as PDF-based textbooks or simple electronic textbooks. Second-generation digital textbooks, on the other hand, include interactive elements and cover a more extensive range of content than printed textbooks, representing a new form of textbook. They are known as cyberbooks, I-textbooks, hybrid textbooks, or collaborative digital textbooks.

Digital textbooks are characterized by their inclusion of various features. In addition to the digitalized content of existing textbooks, they incorporate diverse user elements, multimedia functions, and communication features. User elements include the ability to highlight important text or take notes. Furthermore, they extend learning by allowing users to look up word definitions, search for further information, and engage in in-depth study through hyperlinks [9]. Multimedia functions are a core feature of digital textbooks, encompassing not only photos but also videos, 360°/3D images or videos, and audio materials that traditional textbooks cannot offer. The inclusion of recorded audio materials and Text-to-Speech (TTS) functions also makes these textbooks accessible to students with visual impairments [5]. Recently, digital textbooks have incorporated AR or VR technologies [7]. Communication functions refer to the features that facilitate interaction between teachers and learners or among learners [11], allowing for active collaboration in an online environment through posting and commenting on discussions or real-time communication. Features that allow students to upload and receive feedback on assignments enable them to comfortably complete and submit their work [19]. 

**Figure 1:**
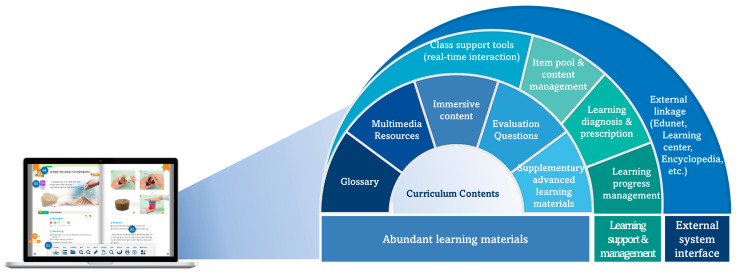
The concept diagram of the digital textbook [18].

In Korea, where national policies are being implemented to digitize textbooks, the term “electronic textbook” was used until 2007, after which the term “digital textbook” was first introduced by the Korea Education and Research Information Service. Ref. [20] defined digital textbooks as “digital learning materials that digitize existing printed textbooks, incorporating the advantages of printed books along with additional convenience features such as search and navigation, as well as multimedia learning functions like animation and 3D, to maximize convenience and learning effectiveness.” This definition has been widely used to date. Figure 1 is a conceptual diagram representing digital textbooks, including not only learning materials but also various additional features.

Recently, efforts have been made to integrate advanced AI technology into digital textbooks. AI can facilitate self-directed learning by providing answers to questions that arise during the learning process, offering customized problems suited to the learner’s level, allowing learners to assess their understanding or engage in more in-depth study. This integration of AI into digital textbooks can promote a significant advancement in personalized and interactive learning. South Korea has announced plans to introduce an open learning platform from 2025 after a two-year development and preparation period from 2023 and to officially use AI digital textbooks for elementary school students in grades 3 and 4, middle school students in grades 1 and 2, and high school students in grades 3 and 4 by 2027, focusing on mathematics, English, and information sciences subjects [21].

### 2.2. Digital Textbook and Sustainability of Education

A sustainable education system offers environmental, economic, and social sustainability to provide educational opportunities for both current and future generations. As an integral component of education, the medium, specifically textbooks, also needs to be designed and chosen with a sustainability perspective. Digital textbooks have many advantages in this regard.

Ref. [6] reviewed various contributions of digital textbooks to sustainability, emphasizing the environmental advantages of electronic books over traditional paper books. Specifically, it mentioned the benefits of reducing energy consumption in classrooms and conserving resources, as well as the potential for economic cost savings by using e-books.

Digital textbooks also offer advantages from the social perspective of sustainability. Refs. [22,23] summarized that digital textbooks can contribute to enhancing students’ awareness of sustainability. Ref. [5] mentioned that for visually impaired students, the use of recorded voices in e-books is possible, implying that digital textbooks can provide equal educational opportunities. 

Moreover, digital textbooks can rapidly adapt and update in response to changes in environments and advancements in modern technology. They can incorporate a variety of technologies and features, and this adaptability can contribute to maintaining the sustainability of education even amidst environmental changes. For example, during the sudden and unexpected shift away from in-person education due to COVID-19, digital textbooks were able to provide sustainable education.

### 2.3. Educational Effectiveness of Digital Textbooks: Focusing on Cognitive Abilities

Textbooks are one of the most crucial mediums for both instructors and learners in the educational field. Therefore, ever since the advent of digital textbooks, their impact on education has been actively researched. In particular, the educational effectiveness of digital textbooks and their various features compared with traditional printed textbooks or static PDF files has been a significant consideration in the adoption of digital textbooks in educational settings.

Many prior studies have reported the positive impact of digital textbooks on students’ cognitive abilities [4,17,24,25,26,27,28]. Improvements in academic achievement in subjects such as English [4], mathematics [17,24], and science [25,26] due to the use of digital textbooks have been documented, as well as enhancements in cognitive competencies such as self-directed learning skills [25,27] and problem-solving abilities [27,28]. Ref. [26] attributes these positive effects to features included in digital textbooks, such as self-testing, highlighting, and generative strategies, which are not present in printed media.

However, some argue that the actual effectiveness of digital textbooks is still uncertain [13,14,29]. Ref. [14] pointed out in a study on students’ preferences for digital versus paper textbooks that while students’ preferences may have a limited impact on actual usage decisions, digital textbooks might not meet students’ expectations. As student preferences for digital textbooks show conflicting results in studies [17], further research is needed to understand how these preferences affect actual continued use intentions or the effectiveness of use. Ref. [29] suggested that digital textbooks may be underutilized and limited in effectiveness due to teachers’ lack of knowledge and skills in using information technology. On the other hand, in a survey of university students, ref. [13] found that the usability of digital textbooks is high, but the effectiveness of digital textbooks has not been sufficiently proven to be fully adopted. It is natural that different studies show different results depending on the specific method, target, and period of use of digital textbooks. Thus, it is important to conduct a longitudinal study to obtain comprehensive and general implications.

### 2.4. Digital Textbook and Affective Competencies: Self-Efficacy, Motivation, and Attitude

Affective competencies, unlike cognitive competencies, are competencies related to the emotional domain, including personality, attitudes, values, as well as motivation, interest, and self-concept towards learning. In the international comparative study TIMSS, the sub-domains of the student attitude toward learning include student readiness to learn, student motivation, student self-concept, and student characteristics [30]. Competencies within the affective domain, such as self-efficacy, intrinsic learning motivation, and learning attitude, are crucial not only on their own but also as they influence academic achievement and act as significant predictors of academic success [31,32,33,34,35].

Self-efficacy refers to an individual’s confidence in their ability to organize and execute the actions necessary to produce a specific outcome [36]. Self-efficacy is known to impact academic achievement significantly [31,32]. Students with high self-efficacy tend to employ more cognitive and meta-cognitive strategies and persevere through challenges, leading to higher academic achievement compared with those with low self-efficacy. In the digitalized educational environment, self-efficacy remains a critical element. Ref. [37] reported that students’ self-efficacy and self-directed learning abilities improved when using e-books equipped with a learning management program. Similarly, ref. [5] noted that the use of e-books positively affects students’ self-efficacy and learning motivation.

Intrinsic learning motivation and learning attitude are also known as crucial affective elements impacting student achievement. Learning motivation is one of the significant predictors of academic achievement, with an increase in motivation known to enhance academic success and learning effectiveness [33,34,35]. In digital educational environments, ensuring that students are continuously motivated and actively engaged in learning is an important challenge [12]. Therefore, there has been considerable research on the variables or methods that can enhance learning motivation and attitude [5,17,19,38].

Ref. [19] reported that utilizing e-books integrated with information technology in the classroom can enhance students’ learning motivation and promote self-directed learning. In [38], students using interactive iBooks responded that their learning motivation and interest had increased. Ref. [17] indicated that compared with static, simple PDF textbooks, e-books, including various features, have a more positive impact on students’ learning motivation.

Numerous prior studies addressing the impact of e-books on affective competencies have been conducted in higher education institutions. In contrast, there has been a relative lack of research on the impact of digital textbooks on affective competencies in primary, middle, and high school learning environments where the role of textbooks is more emphasized. Ref. [5] reported that while e-books had a positive effect on the learning motivation of college students, they did not have the same effect on early school years.

### 2.5. Digital Textbook and Social Competencies: Communication, Collaboration, and Sociability

Social competency is essential for functioning as a member of society, and with the shift to online social interactions following COVID-19, the ability to perform social tasks regardless of environment has become increasingly important. Social competency includes a range of skills necessary for individuals to interact socially and accomplish social tasks [39,40]. Sub-elements of social competency include interpersonal communication skills, collaborative abilities, and sociability [41,42].

Due to their various interactive features, digital textbooks are expected to enhance students’ social competencies compared to simple e-books. As indicated by [43], digital textbooks not only facilitate interaction between students and the e-book but also enable bidirectional communication between students and teachers, thereby potentially increasing the effectiveness of learning. Moreover, these features can promote communication and collaboration among students and enhance their social competencies [44,45]. Ref. [26] reported that the use of e-books increased interaction among students. Ref. [11] confirmed the usefulness of digital technology for communication and collaboration in learning environments through qualitative research with teachers. Ref. [19] noted that the features included in e-books increase pupil–teacher interactions, enriching learning methods and positively impacting academic performance.

However, there are critiques that achieving social competencies can be challenging due to the spatial limitations of digital learning environments and the constraints of media mediating interactions [46,47]. Particularly in online education environments, despite students’ and instructors’ awareness and efforts toward social competencies, there are environmental constraints that prevent adequate interpersonal interactions [48,49]. In such contexts, the importance of interaction features in digital textbooks is more emphasized, necessitating research into the impact of digital textbooks on social competencies.

### 2.6. Nationwide Digital Textbook Policies

With the digitalization of school education, the digitalization of textbooks has also emerged as a policy-critical issue. Even before the rapid digitalization of education due to COVID-19, digital textbooks have been consistently developed and targeted for national adoption in various countries due to their wide-ranging advantages [50].

In 2018, the United Kingdom established LendED, an Edtech (Education Technology) open platform, with support from the British Educational Suppliers Association (BESA) [51]. This platform enables schools to search for and purchase digital educational materials. Singapore has been piloting digital textbooks for first-year students in secondary schools since 2000, and a similar initiative was conducted in Malaysia in 2002. Germany launched the DigitalPakt Schule project in 2019, with the goal of establishing digital infrastructure nationwide by 2024 [52]. In the United States, the federal government has developed the National Educational Technology Plan, providing guidance on technology utilization in U.S. education and focusing on addressing digital education disparities at the state level [53]. Japan, through the “GIGA school project”, has been promoting digital infrastructure and transitioning to digital education through e-learning platforms from 2020 to 2023 [54]. Estonia has emerged as a rising star in European education, utilizing digital learning materials known as “e-schoolbag” and providing digital textbooks free of charge in schools since 2018 [55].

Since 2007, Korea has been developing digital textbooks through a nationwide initiative, attempting to incorporate them into the educational curriculum around 2012, primarily in subjects like social studies, science, and English. Currently, digital textbooks for social studies, science, and English are well developed and distributed in elementary schools (grades 3–6) and middle schools (grades 1–3), and those for English in high schools, and they are actively used. Furthermore, after a two-year preparation period starting from 2023, an open learning platform will be introduced in 2025, initially targeting grades 3–4 in elementary schools and grade 1 in middle and high schools, focusing on subjects like mathematics, English, and IT, with the plan to sequentially and officially adopt AI digital textbooks by 2027.

Before officially adopting digital textbooks nationwide, Korea conducted pilot school projects in selected elementary, middle, and high schools to test the use and effect of digital textbooks in classes. Over three years, the project involved 81 elementary schools and their students in grades 3–6, with digital textbooks for social studies, science, and English subjects. Out of these, 35 schools (1418 students) were selected through random cluster sampling, and a wide range of panel data was collected through a survey. By analyzing these data, this research aims to verify the long-term effects of digital textbooks in affective and social domains.

The pilot school project started before the completion of the digital textbooks’ development, leading to a scenario where students who started the project in grade 4 had to revert to printed textbooks in their third year in the project due to the unavailability of grade 6 digital textbooks. In contrast, students who started using digital textbooks in grade 3 continued to use them until grade 5. Therefore, a comparative analysis between the two groups with controlled usage of digital textbooks is available. This allows for a clearer understanding of the unique effects of digital textbooks compared with those of printed textbooks.

## 3. Methods

### 3.1. Participants

The data analyzed in this research were collected through the longitudinal study on the “Use and Effectiveness of Digital Textbooks in Korea” [56]. Before full-scale nationwide implementation, longitudinal data were collected over three years from 35 digital textbook pilot elementary schools across the country to empirically verify the effectiveness of digital textbook utilization. The study involved 1418 elementary school students participating in social studies and science classes using digital textbooks, consisting of 708 students in the 3rd grade and 710 in the 4th grade. Each student panel participated in a longitudinal survey once a year for a total of three times.

The 3rd grade panel responded up to the 5th grade, and the 4th grade panel responded up to the 6th grade; however, since digital textbooks were only developed up to the 5th grade at the time of the project, the 6th grade students in the third year participated in the survey without using digital textbooks. That is, the 3rd grade panel became the treatment group that consistently used digital textbooks for three years, while the 4th grade panel became the control group that stopped using digital textbooks after two years. This panel design allows for the understanding of not only the effects of the continuous use of digital textbooks for three years but also the changes when use is discontinued. The composition of the study subjects is shown in Table 1.

### 3.2. Measurement Tools

The measurement tool used in this study was developed based on the “Effectiveness Measurement Tool Development Study” [57] conducted prior to the first year of research [56]. The tool developed in [57] was reported to have satisfactory reliability and validity, and it was refined for use in this study. Based on the measurement tool from [57], a review of the literature on digital textbook effectiveness and its measurement tools was conducted to develop the draft. After this review, the draft was refined through two rounds of expert Delphi surveys and reviews by field teachers, followed by a preliminary survey for validity and reliability analysis culminating in the development of the final questionnaire. 

The affective and social competencies analyzed in this study were measured with items that respondents answered on a 5-point Likert scale indicating their agreement with the statements. The composition of the survey, representative items, and reliability are shown in Table 2. Full questionnaires are presented in Appendix A and Appendix B.

Affective competencies were measured as sub-competencies of self-efficacy, learning motivation (intrinsic), and learning attitude. Self-efficacy was measured with three items reflecting the belief in one’s ability to successfully complete difficult tasks. Learning motivation consisted of intrinsic and extrinsic motivation. This study measured intrinsic motivation through three items, asking about interest and satisfaction with the learning content. Learning attitude was measured with four items inquiring about participation in class and individual study, such as diligent participation in class time and earnestly doing homework, preparation, and review.

Social competencies were measured as sub-competencies of communication skills, collaboration skills, and sociability. Communication skills were measured with six items as follows: three on the ability to listen and understand others’ stories and intentions and three on interaction skills. Collaboration skills were measured with six items as follows: three on the ability to respect each other and three on sharing information and mutual feedback when performing joint tasks. Sociability was measured with three items, each on interpersonal relationships and sociability.

The reliability of the measurement tools for these research variables was very high, above 0.85, and the descriptive statistics by year are shown in Table 3.

### 3.3. Research Design and Data Analysis

The study panel comprises two groups of students as follows: one group used digital textbooks continuously for three years (starting from grade 3), while the other group used digital textbooks for two years and then switched back to paper textbooks in the third year (starting from grade 4). This study aimed to examine the effects of using and discontinuing digital textbooks by comparing the changes in affective and social competencies between the continuous group and the discontinued group. If digital textbooks are considered a treatment, the following research design (Table 4) is possible: the continuous use group is the treatment group, and the discontinued use group is the control group.

The aim of the research design is to compare changes in the affective and social competence between two groups over a three-year period. Test 1 assessed group differences in the change from the first year to second year. If the digital textbook were effective, both groups should show improvement. The analysis of interest was test 2, which compares changes from the second year to third year to test for differences in the effects of continued versus discontinued use. If the treatment group showed higher improvement than the control group in test 2, it could be concluded that the digital textbook is effective.

The analysis involved a panel regression analysis. A random-effect panel regression was conducted in this study using the Hausman test, and considering the hierarchical structure inherent in schools, a clustered-robust standard error was estimated by setting the schools as a cluster to improve the accuracy of statistical decision making. When inputting the time variable, which represents the number of years, into the model to test for two comparisons, the author divided it into two categories to capture the extent of change in each. This is demonstrated in the following Equation (1).

(1) $$y_{it}=\alpha +\beta_{1}\left({D1}_{year,t}\right)+\beta_{2}\left({D2}_{year,t}\right)+\beta_{3}({Group}_{it})+\beta_{4}\left({D1}_{year,t}\times {Group}_{it}\right)\;+\beta_{5}\left({D2}_{year,t}\times {Group}_{it}\right)+e_{it},$$

where $y_{it}$ is the cognitive ability of student i in time t, and the five independent variables are ${D1}_{year,t}$, ${D2}_{year,t}$, ${Group}_{it}$, ${D1}_{year,t}\times {Group}_{it}$, and ${D2}_{year,t}\times {Group}_{it}$, respectively. ${D1}_{year,t}$ is −1, 0, and 0 if *t* is 1, 2, and 3, respectively; ${D2}_{year,t}$ is 0, 0, and 1 if *t* is 1, 2, and 3, respectively. ${Group}_{it}$ is 0 if student *i* is in the treatment group and 1 if student *i* is in the control group. $\beta_{1}$ represents the estimated value for the pre- and post-difference (first year to second year) in the control group, while $\beta_{2}$ signifies the estimated value for the pre- and post-difference (second year to third year). $\beta_{3}$ indicates the difference in abilities between the treatment and control groups in the baseline year (second year). By adding the interaction terms of year dummy variables and the group variable to this equation, the longitudinal effects of digital textbooks can be examined. The regression coefficient of the interaction terms, $\beta_{4}$, represents the difference in the change between the treatment and control groups from the second year, while $\beta_{5}$ indicates the difference in the change between the groups from the third year.

This study used longitudinal data measured over three years, and to reflect this in the analytical model, two “year dummy variables” were created as ${D1}_{year,t}$ and ${D2}_{year,t}$. Table 5 shows the research design and the variables for each test.

With these settings, in the above equation, $\beta_{1}$ is the estimate of the pre- (first year) to post-difference (second year) in the control group, and $\beta_{2}$ is the estimate of the pre- (second year) to post-difference (third year). $\beta_{3}$ denotes the difference in competency between the treatment and control groups in the second year, which is the baseline year. By adding an interaction term between the year dummy variable and the group variable in this equation, the author can test the longitudinal effect of the digital textbook. The regression coefficient of the interaction term, $\beta_{4}$, indicates the difference in the amount of change in the second year between the treatment and control groups, and $\beta_{5}$ indicates the difference in the amount of change in the third year between the treatment and control groups.

Specifically, the effect of digital textbooks, which is the research hypothesis of this study, can be verified through the statistical significance of $\beta_{5}$, which is the difference in the change in the third year (test 2). This is equivalent to a difference-in-difference (DID) estimate, which is commonly used to test the effectiveness of policy programs [58,59]. DIDs are often used to compare differences between treatment and control groups before and after a specific event. By setting up a treatment group and a homogeneous control group to compare the before-and-after relationship, unobservable factors that vary over time are removed, which has the advantage of identifying causal effect estimates. In other words, $\beta_{5}$ means the difference between the treatment group and the control group in the change in the third year after controlling for heterogeneity between the groups in the first year. Therefore, it is possible to verify the effect of continued use of digital textbooks by estimating $\beta_{5}$.

## 4. Results

Unlike the treatment group, which used digital textbooks in all three years, the control group did not use digital textbooks in the third year. The author uses panel regression analysis to compare the longitudinal changes in affective and social competencies between the treatment and control groups.

### 4.1. Effect of Digital Textbooks on Affective Competencies

The results of the panel regression analysis of changes in affective competencies are shown in Table 6.

**Figure 2:**
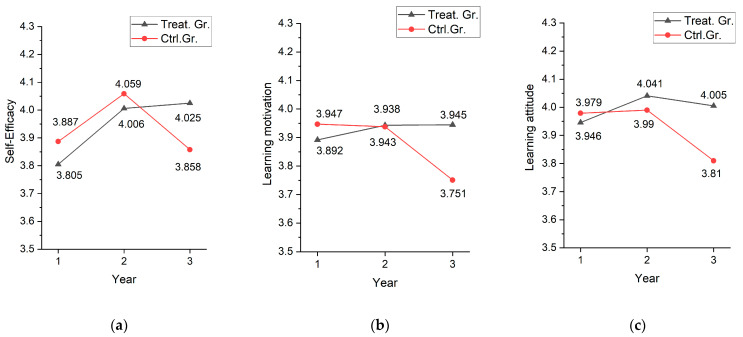
Group differences in the change in affective competencies as follows: (**a**) self-efficacy; (**b**) learning motivation; (**c**) learning attitude.

First, for differences in year-to-year changes in self-efficacy between the continued use group (treatment group) and the discontinued use group (control group), there was no difference between the groups before the second year (b = 0.029, *p* > 0.05). However, the difference in year-to-year changes in self-efficacy was significant in the third year (b = 0.220, *p* < 0.001). There was no difference between the groups in the first and second year of using the digital textbooks, but in the third year, when the control group did not use the digital textbooks, the continuous use group had a 0.220-point increase in self-efficacy. The result was similar for learning motivation, where continuous use of digital textbooks by the third year led to increased mean learning motivation by 0.189 points compared with that of the control group. However, in the case of learning attitudes, the treatment group improved more in the second year (b = 0.144, *p* < 0.01), but since both groups were using digital textbooks at this point, this result cannot be attributed to the effect of using digital textbooks but rather to differences in characteristics between the groups. The interaction term, (${D2}_{year,t}\times {Group}_{it}$) for the change from second year to third year, which represents the difference in the use of digital textbooks, showed that the treatment group had a 0.144 improvement in learning attitude compared with that of the control group, which was statistically significant. The result shown in Table 6 is visualized in Figure 2.

Figure 2 shows that the treatment group, who used digital textbooks for three years, either increased or maintained their affective competencies, while the control group’s affective competencies tended to decrease with the discontinuation of digital textbooks.

### 4.2. Effect of Digital Textbooks on Social Competencies

The results of the panel regression analysis of changes in social competencies are shown in Table 7.

**Figure 3:**
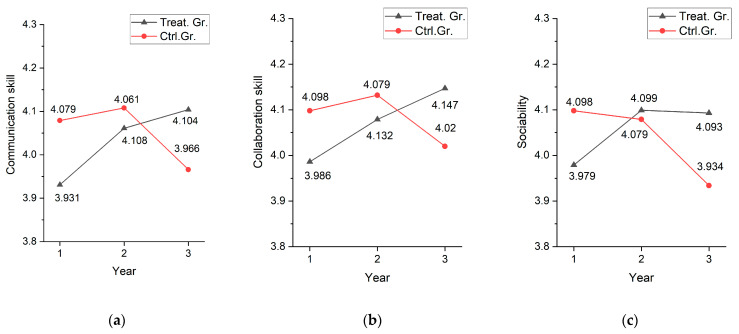
Group differences in the change in social competencies as follows: (**a**) communication skill; (**b**) collaboration skill; (**c**) sociability.

Communication skills improved for both groups in the second year, with no significant difference (b = 0.101, *p* > 0.05), but in the third year, communication skills decreased for the control group and improved for the treatment group, with a significant difference of 0.185. Similarly, for collaboration skills, there was no difference between groups in the second year (b = 0.059, *p* > 0.05), but in the third year, the treatment group showed higher improvement in collaboration skills (b = 0.180, *p* < 0.01). For sociability, the treatment group made higher improvement in both the second and third year. The difference in the second year, when both groups used the textbook, could be attributed to unexplained between-group differences, but the difference in the third year, when only the treatment group used the textbook, controlled for between-group differences in the first year, suggesting an effect of using the textbook. Thus, the continuous use of digital textbooks for more than two years had a significant impact on the improvement of social competencies. Figure 3 is a diagram of the between-group differences in the change in social competencies based on the panel regression estimates.

The communication and collaboration skills of the treatment group tended to improve continuously with the use of digital textbooks, while sociability showed a pattern of improvement in the second year and was maintained thereafter. On the other hand, the control group showed higher social competencies than the treatment group in the first year, with little change in the second year and a sharp decline in the third year when digital textbooks were not used. These results suggest that the continuous use of digital textbooks is effective in improving social competencies and that discontinuing their use may lead to a decrease in social competencies.

## 5. Discussion

This study analyzed differences in the changes in affective and social competencies between a panel of 1418 primary school students who used digital textbooks continuously for three years and those who stopped using digital textbooks. A panel of 710 fourth grade students used digital textbooks for two years and then returned to paper textbooks in the third year; they were considered to be the control group. A panel of 708 third grade students continued to use the digital textbooks until the third year; they were considered to be the treatment group. The research hypotheses are as follows:

**Hypothesis** **1 (H1).** *There will be a difference in the change in affective domain competencies (self-efficacy, learning motivation, and learning attitude) between the group that continued using digital textbooks and the group that stopped using them.*

**Hypothesis** **2 (H2).** *There will be a difference in the change in social domain competencies (communication skills, collaboration skills, and sociality) between the group that continued using digital textbooks and the group that discontinued their use.*

The results show significant improvements in affective and social competencies in the treatment group compared with those in the control group, suggesting that digital textbooks have the effect of improving affective and social competencies. Therefore, H1 and H2 are supported. These results can be interpreted in various contexts.

### 5.1. Effect of Digital Textbooks on Affective Competencies

The sub-competencies of the affective domain analyzed in this study are self-efficacy, learning motivation, and learning attitudes. The result shows that the continuous use of digital textbooks has a positive effect on increasing students’ affective competencies compared to paper textbooks. These findings are in line with previous studies that reported the positive impact of digital textbooks on students’ self-efficacy [5,37] and learning motivation [5,17,19,38,60] during the learning process. Especially, affective competencies are important because they can act as a mediator and bring a positive impact on learning outcomes [60] or the intent to use the digital textbook.

The interactivity of digital textbooks can be considered to be a factor that makes digital textbooks enhance affective competencies. It has been reported that the rich interactivity of e-books enhances affective competencies like learning motivation [61] and learning attitude [62]. These roles of the interactive features of digital textbooks also act meaningfully in social competencies. 

### 5.2. Effect of Digital Textbooks on Social Competencies

This study also explored the impact of using digital textbooks on the social domain competencies of communication, collaboration, and sociability. These competencies are often referred to as the core competencies of the 21st century and are considered to be important in many studies. The result shows that digital textbooks improve students’ social competencies compared with paper textbooks. These results support the findings of previous studies such as [63].

The various communication features offered by digital textbooks can be considered to be a reason why digital textbooks can improve students’ social competencies. Ref. [26] cited features such as self-testing, highlighting, and generative strategies included in digital textbooks, unlike in print media, to be reasons why digital textbooks can improve students’ cognitive competencies. As for social competencies, the various usability elements of digital textbooks can facilitate interpersonal interaction by providing connectivity. Digital textbooks include windows that mediate teacher–pupil interactions as well as pupil–pupil interactions. These features allow students to interact actively, share knowledge, and collaborate. Ref. [64] found that digital learning materials stimulate students to share their knowledge with others actively. A qualitative study by [11] also found that the use of digital technologies in blended learning environments increased collaboration among students. When utilized appropriately, the wide range of digital technologies and features that are included in digital textbooks appear to enhance students’ communication, collaboration, and sociability by increasing interpersonal interaction.

In addition, the result shows that the control group experienced a significant decrease in their communication and collaboration skills when they returned to using only paper textbooks in the third year. This can be attributed to the fact that the interactions mediated by digital textbooks decreased with the cessation of digital textbook use. Previous studies exploring the effectiveness of digital learning materials have shown that digital learning materials have the advantage of being location-independent, i.e., students can give and receive sustainable feedback not only when they are in the classroom but also when they are at home [65,66]. In other words, digital learning has continuity and sustainability [67], and students are more likely to engage in social activities and develop social competence because of this when using digital textbooks compared with paper textbooks. It can be argued that the discontinuation of digital textbooks in the control group undermined the positive effects of the persistence and flexibility of digital textbooks.

### 5.3. Factors Affecting the Effectiveness of Digital Textbooks

This study focused on primary school students in grades 3–6, finding that the continued use of digital textbooks had a positive impact on affective and social competencies. In addition to primary school students, there is the active literature on the effectiveness of digital textbook research in a variety of age groups.

A meta-analysis study exploring the impact of e-books on mathematical achievement found that the effect of e-books was higher in pre-schools than in higher education institutions, suggesting that the younger the age the more proficient the digital skills [8]. This result is in line with the result of [1]. However, in an experiment with university students, ref. [5] found that e-books had a positive effect on motivation to learn but not in the early school stages [68], suggesting that university students were more responsive and active in using e-books.

From these results, it seems that the effectiveness of using digital textbooks is influenced by the proactivity and flexibility of the users. In this context, many studies report that the effectiveness is affected by factors like the digital literacy of students or teachers. Moreover, the actual usage of digital textbooks is known to be affected by factors such as perceived effectiveness [7] and perceived ease of use [69]. In this study, there was no significant difference in the control group’s learning motivation and social skills in the first and second years even though they used digital textbooks. This result can be attributed to differences in initial characteristics between the groups, but a further interpretation could be attributed to other uncontrolled factors such as that the control group was less flexible in adopting digital textbooks or that there were differences in the extent to which teachers utilized digital textbooks.

While the main finding of the study is that the long-term, sustained use of digital textbooks is effective for affective and social competencies, there are also detailed findings worth noting. In the group that used digital textbooks for three years, there was an increase in competencies between the first to second year and the second to third year, but the slope tended to decrease somewhat in the second to third year. Similarly, ref. [8], in a meta-analysis of 17 quantitative studies, reported increased effectiveness when e-books were used for shorter periods of time (less than four weeks). While the study did not directly analyze the reason, it did suggest that e-books as well as all novel attempts at learning can lose their appeal over time and that teachers should consider this when introducing e-books into the classroom. It is also important to ensure that e-books are constantly updated and improved to maintain their effectiveness. South Korea has been systematically improving its e-textbooks by adopting digital technologies, and e-textbooks with AI technology are now being developed for introduction in 2025, which is expected to help maintain the effectiveness of e-textbooks.

### 5.4. Limitations and Further Research

This study found that digital textbooks had a positive effect on students’ affective and social competencies. Along with many previous studies that have shown positive effects on students’ cognitive competencies, this study supports the effectiveness of digital textbooks in affective and social competencies. Despite the significance of this study, there is a limitation as follows: it only proves the effectiveness of digital textbooks and does not identify the cause of this effect. Therefore, further qualitative research is needed to identify the causes or mechanisms (e.g., mediators) by which digital textbooks have these effects and to explore ways to enhance the effectiveness of digital textbooks.

In addition, this study used students’ self-reported survey results as a method of assessing their competencies. While this method facilitates the assessment of students’ perceived competence, it does not allow for the comparison of objective competencies. Therefore, the author recommends further research to collect responses from teachers as well as students or to use a standardized test instrument. 

Moreover, the measurement tools used in this study are an advancement of the one developed through prior research [60], which reported satisfactory reliability and validity for the measurement tools. However, this study still has a limitation regarding the measurement tools since no separate validity verification was conducted after modifying the tools.

Finally, this study is significant in that it analyzes a unique research case of South Korea’s digital textbook pilot schools, which is a comparative study that will be difficult to conduct in the future. However, this experimental design has the limitation that it can only identify the effect of discontinuing the use of digital textbooks not the effect of using or not using them. In order to clearly identify the effect of any treatment, it is appropriate to compare the experimental group with the control group and the treatment group with the non-treatment group, but such an experimental design has limitations in the Korean context since prohibiting the use of digital textbooks that have already been developed and released (non-treatment) may infringe on students’ right to learn.

However, such an experimental design may still be feasible for countries or policies where digital textbooks are in the development stage or have not been distributed nationwide, which is the case for AI digital textbooks in Korea. Therefore, considering the limitations of this study, it is necessary to study the longitudinal effects of digital textbooks through a precise between-group experimental design such as by designing a longitudinal effectiveness analysis study in advance and obtaining pre-treatment scores. It is also proposed that the existing research is extended to collect longitudinal data to verify the more long-term effects of digital textbooks.

## 6. Conclusions

Digital textbooks are a learning medium that can quickly adapt to the changes of the times, practice sustainable education, and improve students’ competencies through various functions. This study conducted a longitudinal analysis of the long-term impact of digital textbooks on the affective and social competencies of elementary school students. The result shows that those who used digital textbooks consistently for three years exhibited sustained improvements in affective and social competencies compared with those who stopped using them in the middle of the study. This suggests that continued use of digital textbooks has a positive impact on students’ affective and social competencies. These findings have several important implications.

First, these findings extend the result of existing studies on the effectiveness of digital textbooks, which have been conducted on a small scale, and examine the effects of digital textbooks over a longer period of time and in a larger sample. Previous studies have been limited by relatively small sample sizes and short experimental periods for the design of experimental and control groups. This study maintains the design of the experimental and control groups to clearly show the effect of digital textbooks while analyzing a larger sample size of 1418 participants and data collected over a longer period of three years. The result of this study supports the results of previous studies and contributes to generalizability. This study provides a comprehensive understanding of digital textbooks and provides findings that are immediately applicable to nationwide policymaking.

Second, this study shows a positive effect of digital textbooks not only on cognitive competencies but also on affective and social competencies. The findings of this study have educational significance as affective and social competencies have been highlighted as core competencies in the 21st century and the development of these competencies has been included as one of the main goals of education. In particular, the results show that digital textbooks help to improve a variety of competencies and not only cognitive competencies, thus promoting a focus on the development of students’ various competencies through digital textbooks.

Furthermore, the result shows that the effects were sustained in the group that used digital textbooks consistently and were not sustained when they stopped using them. This emphasizes the importance of the consistent use of digital textbooks. These findings should be taken into consideration when formulating policies on digital textbooks and when teachers and students use them in real-world learning situations. From a policymaking perspective, this study suggests that policy, development, and introduction of digital textbooks should be formulated to ensure continuity. From an empirical perspective, the findings of this study point out that the current school environment, where the continuity of digital textbook usage during lessons varies among teachers, could undermine the effectiveness of digital textbooks. Therefore, to ensure the effective utilization of digital textbooks, it is necessary to not only provide digital literacy education for teachers but also to educate them about the importance of the consistent usage of digital textbooks and to encourage their continuous utilization. Developing and disseminating instructional strategies for utilizing digital textbooks could also be one approach.

In conclusion, the results showed the positive effect of digital textbooks on students’ affective and social competencies. This study broadens the scope of the existing literature on digital textbooks by validating their effectiveness with South Korea’s nationwide data and practically provides policy recommendations and theoretical foundations that can be utilized. Additionally, by demonstrating the effectiveness of digital textbooks in not only students’ academic achievements and cognitive skills but also their affective and social competencies, it contributes to discussions on students’ socio-emotional development in the future digitalized classroom.

## Figures and Tables

**Table 1:** Participants.

Variables	Group	Grade				Total	
		Treatment Group		Control Group			
		Sample Size	Ratio	Sample Size	Ratio	Sample Size	Ratio
Gener	Boys	333	47.0%	351	49.4%	684	48.2%
	Girls	375	53.0%	359	50.6%	734	51.8%
Region Scale	Metro cities	347	49.0%	356	50.1%	703	49.6%
	Medium cities	149	21.0%	144	20.3%	293	20.6%
	Small cities	212	29.9%	210	29.6%	422	29.8%
Total		708	100.0%	710	100.0%	1418	100.0%

**Table 2:** Variables’ description and reliability.

Variables	Sample Item	Item No.	Reliability *
**Affective competencies**			
Self-efficacy	If I try hard, I can understand even difficult material	3	0.856
Intrinsic motivation	I enjoy learning new things	3	0.855
Learning attitude	I participate actively during class	4	0.838
**Social competencies**			
Communication skill	When talking with friends, I consider their perspective	6	0.919
Collaboration skill	When doing group activities, I try to be helpful to my group members	6	0.922
Sociability	I easily make new friends	6	0.897

* Cronbach’s α coefficient.

**Table 3:** The descriptive statistics for each year.

Group	Variable	First Year			Second Year			Third Year		
		N	Mean	SD	N	Mean	SD	N	Mean	SD
Treatment group	Self-efficacy	708	3.81	0.87	708	4.01	0.83	700	4.02	0.86
	Intrinsic motivation	708	3.90	0.80	708	3.86	0.82	700	3.78	0.79
	Learning attitude	708	3.95	0.80	708	4.04	0.78	700	4.00	0.80
	Communication skill	708	3.93	0.78	708	4.06	0.72	701	4.10	0.72
	Collaboration skill	708	3.99	0.84	708	4.08	0.80	701	4.15	0.75
	Sociability	708	3.98	0.82	708	4.10	0.79	701	4.09	0.78
Control group	Self-efficacy	710	3.89	0.82	710	4.06	0.82	706	3.86	0.84
	Intrinsic motivation	710	3.90	0.79	710	3.85	0.78	706	3.63	0.79
	Learning attitude	710	3.98	0.82	710	3.99	0.79	706	3.81	0.77
	Communication skill	710	4.08	0.75	710	4.11	0.75	708	3.97	0.74
	Collaboration skill	710	4.10	0.82	710	4.13	0.80	708	4.02	0.76
	Sociability	710	4.10	0.81	710	4.08	0.80	708	3.93	0.76

**Table 4:** Research design.

Group	First Year	Second Year	Third Year
Treatment group	O	O	O ^(a)^
Control group	O	O	X ^(b)^
	← Test 1: mean difference (second yr.–first yr.) →		
		← Test 2: mean difference (third yr.–second yr.) →	

^(a)^: DT used (digital textbook used), ^(b)^: DT not used (paper textbook used).

**Table 5:** Estimated average differences across years and cohorts.

	1st Year	2nd Year	3rd Year	2nd Year–1st year	3rd Year–2nd Year
${D1}_{year,t}$	−1	0	0		
${D2}_{year,t}$	0	0	1		
Treat.* Gr.**	$\alpha -\beta_{1}+\beta_{3}{-\beta }_{4}$	$\alpha +\beta_{3}$	$\alpha -\beta_{2}+\beta_{3}+\beta_{5}$	$\beta_{1}+\beta_{4}$	$\beta_{2}+\beta_{3}$
Ctrl.*** Gr.	$\alpha -\beta_{1}$	α	$\beta_{1}$	$\beta_{1}$	$\beta_{2}$
				**test 1**	**test 2**
Treat. Gr. − Ctrl. Gr.	$\beta_{3}{-\beta }_{4}$	$\beta_{3}$	$\beta_{4}$	$\beta_{4}$	$\beta_{5}$

*: treatment; **: group; ***: control.

**Table 6:** Group differences in the change in affective competencies.

	Self-Efficacy			Learning Motivation			Learning Attitude		
Variables	b ^(b)^		SE	b ^(b)^		SE	b ^(b)^		SE
${D1}_{year,t}$	−0.172	***	(0.040)	−0.009		(0.040)	−0.011		(0.042)
${D2}_{year,t}$	−0.201	***	(0.030)	−0.187	***	(0.037)	−0.180	***	(0.031)
${Group}_{it}$	−0.053		(0.059)	−0.005		(0.064)	−0.051		(0.053)
${D1}_{year,t}\times {Group}_{it}$	0.029		(0.054)	0.060		(0.063)	0.084		(0.051)
${D2}_{year,t}\times {Group}_{it}$	0.220	***	(0.060)	0.189	**	(0.072)	0.144	**	(0.053)
Constant	4.059	***	(0.051)	3.938	***	(0.052)	3.990	***	(0.051)
N ^(a)^	4245			4245			4245		

**: *p* < 0.01; ***: *p* < 0.001. ^(a)^: number of observations; ^(b)^: regression coefficient.

**Table 7:** Group differences in the change in social competencies.

	Communication Skill			Collaboration Skill			Sociability		
Variables	b ^(b)^		SE	b ^(b)^		SE	b ^(b)^		SE
${D1}_{year,t}$	−0.029	***	(0.040)	−0.034		(0.047)	−0.019		(0.038)
${D2}_{year,t}$	−0.142	***	(0.031)	−0.112	***	(0.038)	−0.145	***	(0.034)
${Group}_{it}$	−0.047		(0.061)	−0.053		(0.058)	−0.020		(0.056)
${D1}_{year,t}\times {Group}_{it}$	0.101		(0.057)	0.059		(0.053)	0.139		(0.049)
${D2}_{year,t}\times {Group}_{it}$	0.185	***	(0.054)	0.180	**	(0.059)	0.139	**	(0.043)
Constant	4.108	***	(0.049)	4.132	***	(0.050)	4.079	***	(0.052)
N ^(a)^	4245			4245			4245		

**: *p* < 0.01; ***: *p* < 0.001. ^(a)^: number of observations; ^(b)^: regression coefficient.

## Data Availability

Data is contained within the article.

## Appendix A

**Table A1:** Questionnaire on the affective competencies of digital textbooks.

	Strongly Disagree	Disagree	Neutral	Agree	Strongly Agree
**Self-efficacy**					
I understand the content well when studying at school	①	②	③	④	⑤
I can understand complex and difficult content	①	②	③	④	⑤
With effort, I can learn difficult content well with effort	①	②	③	④	⑤
**Intrinsic motivation**					
Learning new things is enjoyable	①	②	③	④	⑤
I find it more interesting to learn difficult content than easy content	①	②	③	④	⑤
I am happy to learn things I did not know before	①	②	③	④	⑤
**Learning attitude**					
I concentrate well during class	①	②	③	④	⑤
I participate actively in class	①	②	③	④	⑤
I complete my homework without missing it	①	②	③	④	⑤
I diligently do my preview and review studies	①	②	③	④	⑤

## Appendix B

**Table A2:** Questionnaire on the social competencies of digital textbooks.

	Strongly Disagree	Disagree	Neutral	Agree	Strongly Agree
**Communication skill**					
When talking with friends, I think about how they feel and listen carefully	①	②	③	④	⑤
I pay close attention to their expressions, voice, and gestures to better listen to my friend	①	②	③	④	⑤
I make an effort to understand the meaning behind my friend’s words	①	②	③	④	⑤
I speak by asking if my friend understands what I mean	①	②	③	④	⑤
When talking with friends, I speak considering their perspective	①	②	③	④	⑤
I can express agreement or disagreement with my friends’ thoughts	①	②	③	④	⑤
**Collaboration skill**					
I do not ignore my friend’s thoughts even if they are different from mine	①	②	③	④	⑤
I try to listen even if my friend’s thoughts differ from mine	①	②	③	④	⑤
I believe that everyone’s thoughts have their merits	①	②	③	④	⑤
During group activities, we share our thoughts with each other to complete it successfully	①	②	③	④	⑤
During group activities, I try to help my group friends	①	②	③	④	⑤
During group activities, I share the information I have found with my group friends	①	②	③	④	⑤
**Sociability**					
I get along well with all my classmates	①	②	③	④	⑤
I prefer being with others than be1ing alone	①	②	③	④	⑤
I am good at talking with friends	①	②	③	④	⑤
I make new friends easily	①	②	③	④	⑤
I tend to start conversations when talking with friends	①	②	③	④	⑤
I can easily talk to new friends	①	②	③	④	⑤
